# Assessment of needle bending and tracking requirements for optimized needle placement in combined intracavitary/interstitial gynecologic brachytherapy

**DOI:** 10.1007/s00066-025-02367-2

**Published:** 2025-02-06

**Authors:** Andre Karius, Vratislav Strnad, Michael Lotter, Stephan Kreppner, Ricarda Merten, Rainer Fietkau, Christoph Bert, Claudia Schweizer

**Affiliations:** 1 Department of Radiation Oncology, Universitätsklinikum Erlangen, Friedrich-Alexander-Universität Erlangen-Nürnberg (FAU), Universitätsstraße 27, 91054 Erlangen, Germany; 2 Comprehensive Cancer Center Erlangen-EMN (CCC ER-EMN), Erlangen, Germany

**Keywords:** Image guidance, Adaptive brachytherapy, Intraoperative navigation, Optical infrared tracking, Needle displacement

## Abstract

**Purpose:**

Needle tracking using external prediction techniques such as optical tracking is a modern approach aimed at improving implantation accuracy in gynecologic brachytherapy. This study aims to investigate the corresponding impact of needle bending in situ and to analyze needle path deviations from the intended locations occurring in our current clinical workflow that only considers ultrasound imaging without tracking.

**Methods:**

We developed a semi-automated approach for reconstructing brachytherapy needles based on planning CTs and compared the respective accuracy to the also determined intra-observer variability of manual reconstructions. Based on this, we measured needle bending in situ for 89 patients and calculated the Euclidean distances between actual needle tips and needle tip predictions both longitudinally and laterally to the insertion direction. Furthermore, we compared actual and intended spacings between inserted needles to estimate implantation uncertainties with respect to our current clinical workflow.

**Results:**

Our developed reconstruction featured an accuracy of 0.17 ± 0.08 mm, which was improved compared to the intra-observer variability of 0.21 ± 0.11 mm. Needle bending depended strongly on needle length and ranged from 3.6 ± 2.1 mm for 100–120 mm needles up to 7.9 ± 3.0 mm for 200–220 mm needles. Deflections in the transverse direction were substantially higher than tip deviations in the longitudinal direction. Furthermore, we found deviations from an equidistant spacing between needle paths of 1.4 ± 1.2 mm in the transverse direction.

**Conclusion:**

Inserting brachytherapy needles can be substantially affected by transverse needle bending in situ, which should therefore be corrected for in prediction approaches such as optical tracking.

## Introduction

Combined intracavitary and interstitial brachytherapy is well established for treating gynecologic malignancies such as cervical cancer. “Combined” thereby refers to the intracavitary insertion of a probe into the vagina or uterus in addition to the interstitial implantation of needles for treating bulky diseases or diseases extending to the vagina and/or parametrium [1–5]. This enables dose optimizations during treatment planning concomitant with good clinical outcomes and reasonable toxicity [5–9].

The insertion of probe and needles is performed in most institutions based on ultrasound [1, 2]. This imaging technique provides generally good soft tissue contrast for differentiating tissues in real time. However, especially regions around the fundus uteri or deep in the pelvis may not be sufficiently accessible for corresponding imaging [10–14]. This can lead to the positions of implanted needles deviating from their intended locations or depths.

To address this issue, intraoperative magnetic resonance imaging (MRI) and computed tomography (CT) are increasingly being used to support image-guided interventions [11, 15–17]. In particular, several mobile CT devices such as the Brainlab Airo [18–20] (Brainlab, Munich, Germany) or the ImagingRing [11, 21–23](medPhoton, Salzburg, Austria) ideally suited for brachytherapy have already been launched. However, multiple image acquisitions may be required during respective interventions for adequate needle positioning control. This is associated with effort for medical personnel, e.g., due to the need for lowering the patient’s legs for fitting into the gantry or for leaving the room during imaging, as well as dose exposure to the patient. In this respect, optical tracking approaches exist that aim to track infrared reflective marker tools rigidly attached to the distal needle parts via infrared cameras and, by this rigid relation, to predict the needle course in situ in an initially acquired CT scan [24–26]. A high accuracy of these predictions is thereby important to achieve a geometrically suitable implant, which may be affected by uncertainties associated with the implantation itself, such as needle bending, for instance. An analysis of the corresponding effects seems therefore to be required prior to a clinical implementation of respective systems.

Following these descriptions, two main issues not examined for gynecologic brachytherapy so far have to be considered. First, the impact of needle bending in situ on the respective needle tip predictions has to be examined. Second, the extent of needle path deviations from their intended locations occurring in current clinical workflows should be investigated as a comparative measure. To address these issues, we developed an algorithm for needle reconstructions on CT, based on which needle bending in situ was retrospectively evaluated for 89 patients. Furthermore, a calculation of inter-needle distances served as an estimation of the clinically occurring deviations between actually implanted and intended needle paths.

## Methods

### Clinical workflow

Between 2018 and mid-2023, we treated 89 cervical cancer patients with parametrium and/or vaginal infiltration with a combination of an intrauterine/intravaginal probe and titanium needles. Probes and needles were inserted via a guidance template fixed to the patient under transrectal and transvaginal ultrasound guidance. The template had a thickness of about 1.5 cm in the longitudinal direction, whereby the area in which the needles were actually clamped over a screw cap amounted to approximately 5 mm. Patients were under general anesthesia and in lithotomy position.

**Fig. 1:**
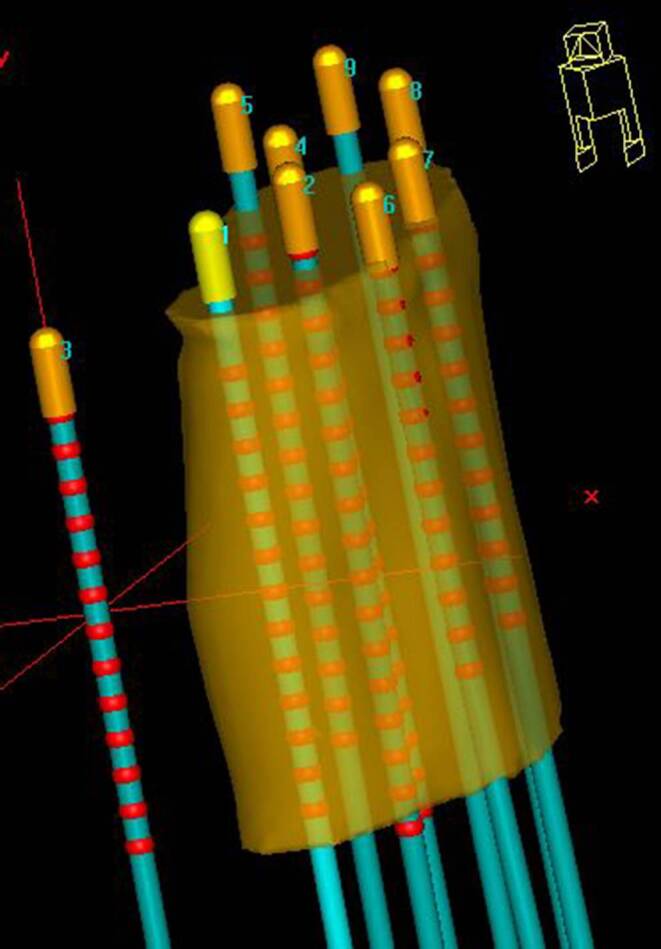
Shown is the needle placement as conducted in an ideal case. The needles (blue) were inserted until their tips (gold) exceeded the contoured high-risk clinical target volume (orange) by up to 9 mm. An exception in this graphic was the intravaginal probe lying outside the contour as shown. Red dots indicate the dwell positions that were defined in this case. Graphic created with Oncentra Brachy (Nucletron, Veenendaal, Netherlands)

Depending on the individual anatomy and tumor localization, we implanted, considering all patients, titanium needles of various lengths ranging from 120 to 220 mm. According to institutional policy, needle tips should have been inserted at minimum up to the cranial border of the high-risk clinical target volume (HR-CTV) [27] identified intraoperatively via ultrasound, or even across this in case of no vessels or intestine being located directly behind and therefore at a risk of being injured (Fig. 1). This is since the most cranial dwell position of an afterloader source can only be located 9 mm away from these tips due to the needle design. After the interventional procedure, patients were transferred to a SOMATOM go.Open Pro CT scanner (Siemens Healthineers, Forchheim, Germany) to acquire a planning CT with applicators in situ, which was reconstructed with a voxel size of about 0.3 × 0.3 × 2 mm^3^. The scan range of this CT was intended to start 1 cm above (cranial direction) the uterus and to end 1–2 cm behind (caudal direction) the distal needle end. The purpose of this procedure was to visualize the entire anatomy of interest as well as the complete needle course and length on the acquired image dataset. Note that the distal needle parts themselves typically protrude 3–8 cm (depending on the patient’s anatomy and needle length) from the template region into the air between the patient’s thighs.

Subsequently, treatment planning was performed using the planning system Oncentra Brachy (Nucletron, Veenendaal, Netherlands). For this, the high-risk clinical target volume (HR-CTV) as identified during surgery was transferred into the CT and contoured following GEC-ESTRO guidelines [12, 27, 28]. Bladder, rectum, intestine, and urethra were considered as organs at risk (OARs). Furthermore, manual reconstruction [29] of the probe and needles within the regions relevant for treatment planning was conducted. Treatment planning itself and treatment delivery are not described here in detail, since they were out of relevance for the present work.

### Semi-automated needle reconstruction

To analyze needle bending, we developed a semi-automated approach for needle reconstruction based on the planning CT in Python. This became necessary, since in the clinical workflow the reconstructions were in most cases limited to the parts needed for treatment planning, as described above. However, to assess the deviations of needle tips from the corresponding straight needle paths as described in Sect. “Needle bending analysis,” considering the entire needle length was required.

**Fig. 2:**
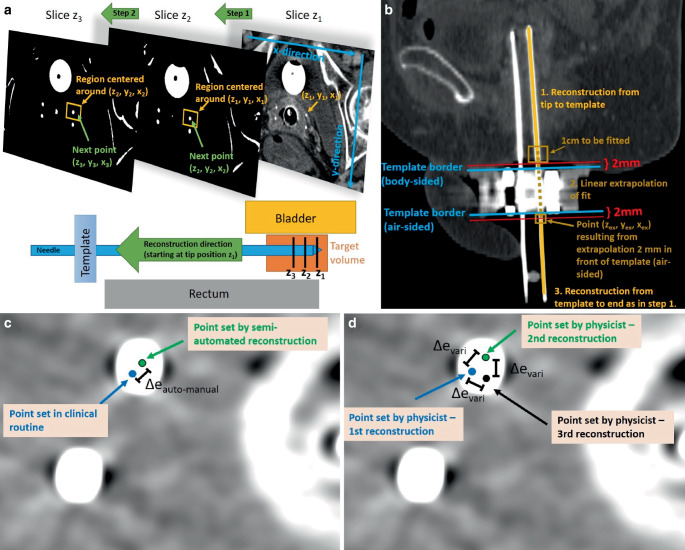
**a** Procedure of our semi-automated reconstruction approach. Starting from the needle tips (z_1_, y_1_, x_1_), we searched in the adjacent CT slice in a region around the tip’s (y_1_, x_1_) coordinate pixel conglomerates with a CT number ≥ 600 HU. The closest conglomerate center was determined to be the next reconstruction point (z_2_, y_2_, x_2_). Starting from this point, the procedure was repeated in the next adjacent slice z_3_, and so on. The schematic representation of the needle in relation to the template and organs at risk clarifies the reconstruction direction used in this approach. **b** Procedure to overcome the reconstruction issues within the template region caused by the occurring strong metal artifacts. At first, the needle was reconstructed as already illustrated in **a** starting from the tip up to 2 mm in front of the template border (body-sided). The last 1 cm reconstructed was then fitted with a straight line, which was extrapolated up to the location 2 mm behind the other template border (air-sided). Starting from the point resulting from this extrapolation, needle reconstruction was again performed as in the first step until the needle end was reached. **c** The quantity $\Updelta e_{auto-\textit{manual}}$ referred to the Euclidean distance between the reconstruction points set by the semi-automatic approach and in the clinical workflow, evaluated in each axial CT slice. **d** For determination of intra-observer variability, needles were manually reconstructed three times and the Euclidean distances $\Updelta e_{vari}$ between the individual points were measured, as illustrated in each axial CT slice

As the starting point for our approach, the tip (z_1_, y_1_, x_1_) as reconstructed for each needle in the clinical workflow was used. From these points, the further reconstruction was automated as follows: In the axial CT slice (longitudinal coordinate z_2_) adjacent to the tip, conglomerates of pixels with a CT number ≥ 600 HU were automatically detected by thresholding within a region of 30 × 30 mm^2^ around (y_1_, x_1_) (Fig. 2a). The threshold of 600 HU was empirically determined to ensure a sufficient delineation of needles from other structures (e.g., calcifications) in each case. The region size of 30 × 30 mm^2^ was selected to consider a sufficient area in case of needle path deviations from the straight z‑line and to ensure a thresholding effect within the region. The centers of all conglomerates were then calculated [30], and the center (y_2_, x_2_) closest to (y_1_, x_1_) (since this referred to the conglomerate most likely related to the examined needle) was considered as associated, adjacent reconstruction point (z_2_, y_2_, x_2_). The same procedure was then conducted in the slice adjacent to z_2_, and the next reconstruction point (z_3_, y_3_, x_3_) was identified to the conglomerate center closest to (y_2_, x_2_). This procedure was conducted until a position 2 mm in front of the template (body-sided) was reached. However, in the template region itself, strong metal artifacts rendered the threshold-based reconstruction impossible.

To overcome this issue, the most caudal 1 cm of the already reconstructed section was automatically fitted with a straight line, which was then extrapolated to the CT slice (z_ex_) located 2 mm behind the template (air-sided; Fig. 2b). The corresponding line point (z_ex_, y_ex_, x_ex_) served as the starting point for the further reconstruction, which was again conducted as above until the needle end was reached. In this way, a reconstruction of the entire needle length was obtained. Needles whose distal parts were cut off on the CT scan, since a too small scan length was chosen during CT acquisition, were reconstructed until this cut-off and considered for the analysis as well.

To evaluate the quality of our approach, we compared the obtained needle paths to the manual reconstructions conducted in the clinical workflow. Considering the anatomical regions where the latter existed as well (see above), we calculated for each axial CT slice the Euclidean distance $\Updelta e_{auto-\textit{manual}}$ between the individual needles reconstructed semi-automatically and manually (Fig. 2c). For comparison, the intra-observer variability of manual reconstructions was determined. Therefore, the reconstruction of 53 needles implanted in 10 randomly selected patients was repeated three times by one medical physicist, and intra-observer variability was then obtained by measuring in each CT slice the Euclidean distances $\Updelta e_{vari}$ between the individual needle locations (Fig. 2d). Both results, $\Updelta e_{auto-\textit{manual}}$ and $\Updelta e_{vari}$, were compared to assess the accuracy of the developed reconstruction algorithm.

### Needle bending analysis

**Fig. 3:**
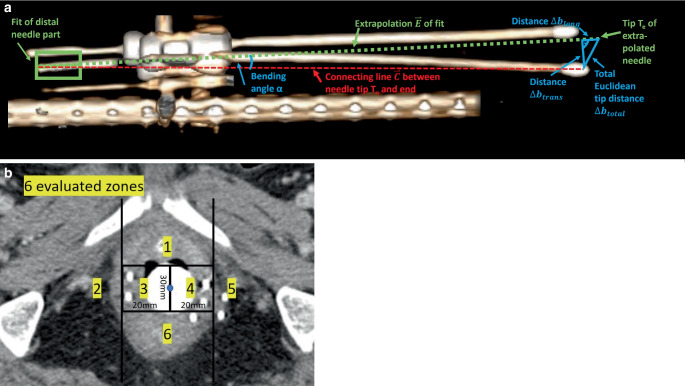
**a** Schematic example for determining the Euclidean deviations between actually reconstructed and predicted needle tips for a needle featuring substantial bending in situ. To obtain the prediction, the most distal 1 cm of the considered needle was fitted with a straight line, which was then extrapolated by the known needle length. The Euclidean deviation to the actual tip was then determined both totally ($\Updelta \mathrm{b}_{\mathrm{total}}$) and separated along the transverse ($\Updelta \mathrm{b}_{\mathrm{trans}}$) and longitudinal ($\Updelta \mathrm{b}_{\mathrm{long}}$) directions (with respect to the predicted course). The bending angle α between the predicted and actual needle courses was determined as well. Graphic created by the RadiAnt Dicom Viewer (Medixant, Poznan, Poland). **b** The patient anatomy was separated into six different segments (1–6) as schematically presented, based on the axial probe midpoint (blue dot) as fixpoint. This separation was conducted in the axial slice containing the longitudinal midpoint of the caudal half of the intrauterine/intravaginal probe as a landmark. For each of these segments, needle bending was evaluated separately to analyze the location dependency of needle bending

Needle bending in situ was evaluated by considering the most distal (located free in air) 1 cm of each semi-automatically reconstructed needle. In cases where the entire needle length was visible on the CT scan, this referred to the most distal 1 cm of the actual needle. In cases where the needle was cut-off on the CT acquisition (since an inadequate scan range was set), the most distal 1 cm of the recorded needle (i.e., the last five 2 mm CT slices starting from the caudal end of the CT image dataset) was considered. This section was fitted with a straight line, which was then extrapolated by the known needle length to obtain a prediction of its tip assuming a straight, non-deflected course. Based on this, we analyzed the bending angle α of the needles as a measure independent of their lengths. This was defined as the angle enclosed by the predicted straight non-deflected needle path $\overset{\rightharpoonup }{E}$ and the connecting line $\overset{\rightharpoonup }{C}$ between the most distal reconstruction point and the reconstructed needle tip, as shown in Fig. 3a. Considering both $\overset{\rightharpoonup }{E}$ and $\overset{\rightharpoonup }{C}$ as vectors in 3D space with magnitudes |$\overset{\rightharpoonup }{C}$| and |$\overset{\rightharpoonup }{E}$|, the bending angle thus results to:

$$\alpha =\mathit{\arccos }\left(\frac{\overset{\rightharpoonup }{C}\circ \overset{\rightharpoonup }{E}}{\left| \overset{\rightharpoonup }{C}\right| \cdot \left| \overset{\rightharpoonup }{E}\right| }\right).$$

Furthermore, we compared the tip prediction to the tip actually reconstructed in the clinical workflow by calculating the corresponding Euclidean distance both totally ($\Updelta b_{\textit{total}}$) and separated for the directions longitudinal $(\Updelta b_{long})$ and transverse $(\Updelta b_{\textit{trans}})$ to the predicted needle course. A schematic illustration of this procedure is provided in Fig. 3a, which shows that the total Euclidean distance $\Updelta b_{\textit{total}}$ could be obtained by measuring the distance between the actual needle tip position T_n_ and the predicted needle tip position T_e_, whereas the longitudinal and transversal deviations $\Updelta b_{long}$ and $\Updelta b_{\textit{trans}}$ were determined via basic trigonometry:

$$\Updelta b_{\textit{total}}=\left| T_{n}-T_{e}\right| ,$$

$$\Updelta b_{\textit{trans}}=\sin \left(\alpha \right)\cdot \left| \overset{\rightharpoonup }{C}\right| ,$$

$$\Updelta b_{long}=\sqrt{\Updelta {b_{\textit{total}}}^{2}-\Updelta {b_{\textit{trans}}}^{2}}.$$

These analyses served as estimation of the impact of needle bending in situ on the corresponding needle paths in situ. The location dependency of this bending was investigated by considering the axial slice containing the longitudinal midpoint of the caudal half of the intrauterine/intravaginal probe as a landmark. In this slice, the patient anatomy was separated into six different segments as visualized in Fig. 3b, and each needle was individually assigned to the segment it passed in this slice. Subsequently, needle bending was evaluated as described above separately for each segment.

### Needle placement uncertainties

**Fig. 4:**
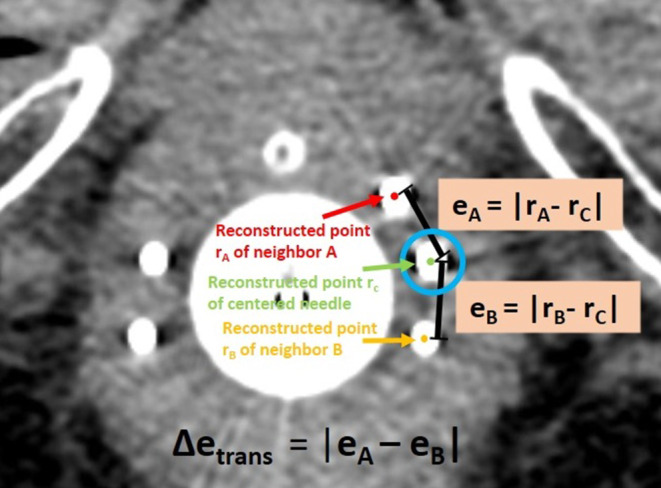
To identify the improvement potential in the transverse plane, we considered needles intended to be placed evenly spaced in between two neighbors, as exemplarily circled blue. The Euclidean distances *e*_*A*_ and *e*_*B*_ to these neighbors were measured, and the resulting discrepancy $\Updelta e_{\textit{trans}}=\left| e_{A}-e_{B}\right|$ served as estimation for the implantation uncertainties in the transverse plane

Furthermore, we investigated the deviations between actually implanted and intended needle locations in situ. For this, we considered all 254 needles that were implanted in between two other needles. To achieve a homogenous implant, these were intended to be evenly spaced with respect to their neighbors, as shown exemplarily in Fig. 4. Deviations from this even spacing were assumed to refer to uncertainties of the implantation procedure and served as an estimation of the deviations between actually implanted and intended needle paths occurring in our clinical workflow.

To determine these deviations, we considered the CT slice containing the first possible dwell position of an examined needle (remember that this is 9 mm away from the tip due to the needle design). In this slice, the Euclidean distances *e*_*A*_ and *e*_*B*_ between the corresponding reconstruction point of the centered needle to the reconstruction points of its neighboring needles *A* and *B* were measured as illustrated in Fig. 4, based on the reconstructions conducted in the clinical workflow. The resulting discrepancy $\Updelta e_{\textit{trans}}=\left| e_{A}-e_{B}\right|$ (Fig. 4) served as measure for the aforementioned implantation uncertainties in the transverse plane.

In this respect, it has to be noted that needles may well deflect differently, and with our method of measuring differences in needle distance, it remains unclear which individual needle actually bends. In particular, if all needles deflect the same amount in the same direction, the tips would seem perfectly aligned but in the wrong position, and even if only the centered needle bends/shifts in a direction perpendicular to the line connecting the two outer needles, no Euclidean discrepancy would be measured. However, in this regard, our two goals of implantation have to be distinguished. The first goal is always to implant needles as close as possible to their intended location, which cannot be assessed retrospectively since the position exactly intended during surgery cannot be redefined on CT data acquired some time ago. In contrast, the second goal is (independent of bending or the aforementioned shifts in any direction) to achieve an equidistant spacing between individual needles for creating a homogenous implant to enable an optimized dose planning based on our clinical brachytherapy experience. This second demand can be analyzed as described above and as conducted in this work, and thus serves as an indicator of uncertainties along the transverse direction associated with our current workflow.

## Results

### Semi-automated needle reconstruction

The developed reconstruction approach revealed a median (± interquartile range) deviation $\Updelta e_{auto-\textit{manual}}$ from the manual reconstructions of 0.17 ± 0.08 mm, with a maximum of 0.46 mm. This was even slightly improved compared to the determined intra-observer variability $\Updelta e_{vari}$ of 0.21 ± 0.11 mm (maximum 0.56 mm). Due to the good reconstruction accuracy, we concluded that our method allowed reliable identification of needle courses for evaluating bending in situ. However, 11 of all 690 needles (1.6%) implanted could not be reconstructed semi-automatically at all and were excluded from our analysis, since their courses merged on the CT and were thus not separable by thresholding. For nine patients and 51 (7.4%) of the 690 implanted needles, the intended scan range of the CT acquisition (Sect. “Clinical workflow”) was not fulfilled and distal parts of the needles were cut-off by a distance of up to 16 mm at maximum, which is small compared to the examined needle lengths. As described in Sect. “Semi-automated needle reconstruction,” these needles were reconstructed until the cut-off, i.e., until the caudal end of the CT image dataset.

### Needle bending

**Fig. 5:**
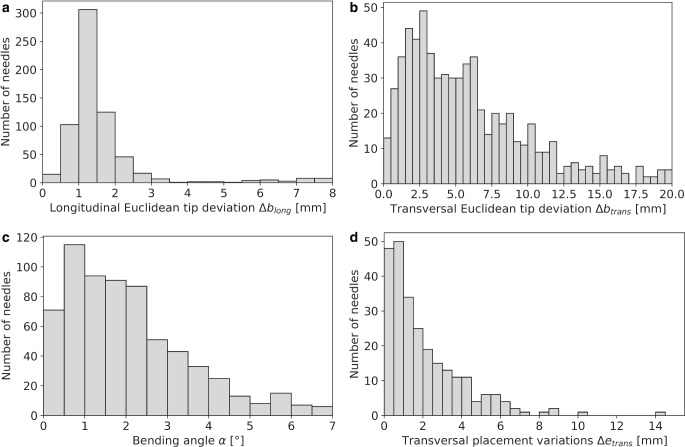
Shown are the Euclidean deviations $\Updelta b_{long}$ and $\Updelta b_{\textit{trans}}$ between actually reconstructed and predicted needle tips for both the longitudinal (**a**) and transverse (**b**) direction with respect to the predicted needle course, respectively. The calculated bending angles between the actual and predicted needle courses are shown as well (**c**). For comparison, the transverse placement deviations $\Updelta e_{\textit{trans}}$ (**d**) obtained for our patients are provided. The results were binned to a bin width of 0.5 mm for distances and 0.5° for the bending angle

**Fig. 6:**
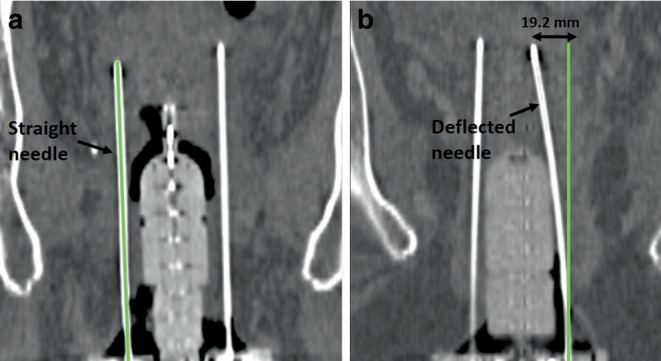
Shown is a visual example of a very small (**a**) and a very large (**b**) needle bending in situ and, hence, deviation between the actual and predicted needle paths. In both cases, a coronal representation of the implant arrangement is provided, and the green line indicates a straight non-deflected needle course (i.e., the tracking prediction). While the predicted and actual courses were almost identical for the first case (**a**), a substantial transverse difference of about 19.2 mm occurred at the needle tip for the second case (**b**)

Considering the 679 reconstructed needles, we found that bending was substantially affected by needle length. For instance, needles with a reconstructed length of 100–120 mm (i.e., considering the interval ]100 mm;120 mm]) showed Euclidean tip deviations $\Updelta b_{\textit{total}}$ from the prediction of 3.6 ± 2.1 mm, whereas deviations of 7.9 ± 3.0 mm were obtained for lengths of 200–220 mm. Independent of the length, tip deviations > 1 mm, > 5 mm, and > 10 mm occurred for 672 (99%), 392 (58%), and 140 (21%) needles, respectively. Discrepancies $\Updelta b_{long}$ along the needle course were with a median of 1.3 ± 0.4 mm in general much smaller than the $\Updelta b_{\textit{trans}}$ of 5.1 ± 3.0 mm found for the transverse direction. The analysis of bending angles revealed a median bending of 1.8 ± 1.1° (range 0.1–6.6°) considering all examined needles, with only small differences between the various needle lengths (e.g., 1.7 ± 0.9° for 100–120 mm needles and 2.2 ± 0.9° for 200–220 mm needles). A detailed report of all findings is provided in Fig. 5 and Table 1, where the high maxima highlighted that bending could lead to severe discrepancies of up to 25.7 mm $\left(\Updelta b_{\textit{total}}\right)$from assumed straight courses. Visual examples of a very small and very large deviations between predicted and actual needle course are shown in Fig. 6.

**Table 1:** Summarized are the Euclidean deviations found between actually reconstructed and predicted needle tips depending on the reconstructed needle length as well as the observed bending angles α. Deviations were calculated both totally $(\Updelta b_{\textit{total}})$ and separated by the directions longitudinal $(\Updelta b_{\textit{total}})$ and transverse $(\Updelta b_{\textit{trans}})$ to the needle course. In each case, the median and interquartile (IQ) range as well as the total range of the results is provided

		Total tip deviation $\Updelta b_{\textit{total}}\:$[mm]		Deviation in longitudinal direction $\Updelta b_{long}$ [mm]		Deviation in transverse direction $\Updelta b_{\textit{trans}}$ [mm]		Bending angles α [°]	
Needle length [mm]	Number of needles	Median ± IQ range	Total range	Median ± IQ range	Total range	Median ± IQ range	Total range	Median ± IQ range	Total range
100–120	31	3.6 ± 2.1	1.2–11.9	1.3 ± 0.4	0.7–5.6	3.2 ± 2.0	0.8–10.9	1.7 ± 0.9	0.4–6.2
120–140	39	4.1 ± 2.0	0.8–12.2	1.4 ± 0.8	0.4–6.1	3.0 ± 1.8	0.6–12.1	1.3 ± 0.8	0.3–5.8
140–160	274	5.7 ± 2.9	0.7–15.3	1.2 ± 0.3	0.2–7.8	4.8 ± 2.8	0.2–15.1	1.8 ± 1.0	0.1–6.2
160–180	243	6.4 ± 3.2	1.0–18.8	1.4 ± 0.5	0.3–7.1	5.5 ± 3.3	0.3–18.6	1.9 ± 1.2	0.1–6.6
180–200	68	6.3 ± 3.1	1.4–18.5	1.5 ± 0.4	0.5–2.6	6.0 ± 3.1	0.9–18.3	1.7 ± 0.9	0.3–5.8
200–220	24	7.9 ± 3.0	1.2–25.7	1.5 ± 0.4	1.1–3.1	7.7 ± 3.1	0.3–19.9	2.2 ± 0.9	0.1–5.7
All	679	5.9 ± 3.0	0.7–25.7	1.3 ± 0.4	0.2–7.8	5.1 ± 3.0	0.2–19.9	1.8 ± 1.0	0.1–6.6

**Fig. 7:**
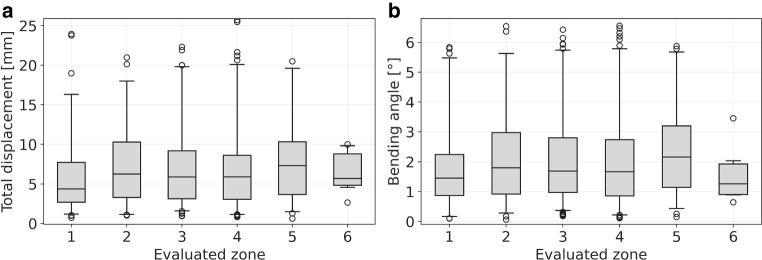
Shown is the deviation between the predicted and the actual needle tip (**a**) as well as the needle bending angles (**b**) that were found for the individual segment zones 1–6. The horizontal lines in the boxes indicate the median, the box the interquartile range, and the whiskers the 95th percentile of the results. Outliers are indicated as circles

In assessing the location dependency of needle bending, we identified 88 (13.0%), 82 (12.1%), 192 (28.3%), 227 (33.4%), 82 (12.1%), and 8 (1.1%) reconstructed needles to be implanted in the segments 1, 2, 3, 4, 5, and 6, respectively. We found that the distributions of the tip deviations between actual and predicted needle tips as well as of the bending angles did not differ substantially between the various regions, as shown in Fig. 7. A slight trend of increased bending in the lateral segments 2 and 5 was observed, with median bending angles of 1.8° (median tip deflection 6.3 mm) and 2.2° (7.3 mm), respectively, compared to an average median of 1.5° (5.5 mm) found for the other segments. Needles implanted in the anterior segment 1 and posterior segment 6 showed, with median angles of 1.5 and 1.3°, respectively, the least bending. However, as mentioned, the differences were small compared to the range of the distributions of the individual results visualized in Fig. 7.

### Needle placement uncertainty

Our analysis of the 254 needles placed in between two neighbors revealed a median placement accuracy $\Updelta e_{\textit{trans}}$ as low as 1.4 ± 1.2 mm (range 0.01–14.7 mm). Only 63 (24.8%), 24 (9.4%), and 2 (0.8%) needles showed corresponding deviations > 3 mm, > 5 mm, and > 10 mm, respectively (Fig. 5). No dependency in needle lengths was found. Hence, needles would have to be implanted with deviations of less than 1.4 mm in the transverse direction from the intended locations to enable benefits at median compared to the current workflow.

## Discussion

In this work, we investigated the impact of needle bending on the corresponding needle path predictions in situ in gynecologic brachytherapy and analyzed the deviations between actually implanted and intended needle paths occurring in our current clinical workflow. While we performed a first corresponding in situ evaluation for gynecologic brachytherapy, needle bending especially for (robot-assisted) prostate brachytherapy has already been investigated albeit using phantoms only [31–35], and corresponding in situ data are currently still lacking for the latter entity as well.

The motivation for the performed investigations was particularly to assess the inaccuracies and uncertainties associated with our current implantation procedures with respect to an evaluation of the potential benefits of recently described [24, 25] novel optical tracking workflows, as mentioned in the Introduction section. The idea of these approaches [24, 25] is to project a predicted needle course in situ in real time onto a single, initially acquired intraoperative CT scan visualized on a screen in the surgical theater, thus allowing intraoperative navigation and guidance of the implantation process without facing the described challenges of multiple repetitive intraoperative CT scans. In this regard, combined tracking camera–CT systems already exist that allow a transfer of the positions of tracked objects in 3D space from the camera coordinate system into the CT coordinate system due to a fixed assembly [24, 25]. If a marker tool attached to the distal end of needles is tracked by the cameras, its position and also the straight course of a rigidly attached needle can thus be displayed in the CT images. The establishment of corresponding tracking workflows is thereby entirely based on the assumption of the aforementioned rigid relationship between the needle tip and the tracked marker tool. Since only this tool but not the needle itself in the patient’s body is recorded by the cameras [24, 25], needle bending cannot be detected with this technique. This means that the predicted needle course (projected onto the CT and used for intraoperative guidance) could deviate from its actual course, causing in a worst-case scenario injury to tissue structures. However, despite being our motivation, it has to be noted that we have not evaluated any optical tracking system in the present work but performed an analysis of needle placement in situ in relation to a general external frame of reference. In this regard, any external approach trying to predict needle courses in situ will be affected by needle bending and potential needle path deviations, independent of an actual technical implementation, although associated uncertainties might vary between various approaches and systems. To assess the implantation uncertainties associated with a routine clinical workflow, a corresponding investigation of needle bending seemed therefore to be required.

In this respect, an accurate placement of needles in the transverse plane is considered important to maximize HR-CTV dose coverage. Although the relationship between the needle locations (and, hence, source positions) and the achievable dose distribution is generally complex and not obvious, creating an equidistant needle spacing was considered to be a good heuristic as a starting point for treatment planning and therefore formed a goal of our implantation procedure. In the present work, we determined deviations $\Updelta e_{\textit{trans}}$ from the intended equidistant positions of only 1.4 ± 1.2 mm, which reflected the high implantation accuracy feasible with ultrasound alone. This was also expected, since the correct targeting of needles can be reliably adjusted using axial ultrasound scans acquired at locations not too deep within the pelvis. The needle deviations of 1.4 mm found for the transverse direction served as an estimation of the maximum uncertainties needle path predictions should feature to be competitive with our current workflow regarding our goal of creating an equidistant spacing between individual needles. This can, in principle, be technically achieved with modern systems, as for instance Koivukangas et al. [36] already showed a decade ago that optical tracking can achieve marker identification accuracies of as low as 0.20 ± 0.10 mm. As already mentioned in Sect. “Needle placement uncertainties,” it must again be noted that our analysis could only evaluate differences with respect to achieving an equidistant spacing but allows no exact statement regarding the deviations between actually implanted and intended needle positions as defined during surgery. This is since if, e.g., all needles deflect by the same amount in the same direction, the tips would seem perfectly aligned but actually be in the wrong position. Nevertheless, our analysis served as estimation of the corresponding implantation uncertainties in the transverse plane associated with our current clinical workflow.

Furthermore, we determined the corresponding impact of needle bending in situ on needle predictions that would result from an extrapolation of needle ends. It has to be noted that the corresponding assessments were performed solely on clinical CT data, since performing additional phantom experiments to compare actual needle bending against the bending estimated from considering the most distal 1 cm of a needle as conducted in this work (to evaluate how accurately and precisely courses can actually be determined by the latter procedure) was beyond the scope of the manuscript. We decided on a consideration of the distal needle ends for this purpose, since there exist corresponding external prediction approaches considering these regions most distant to the needle tips in practical operations [24–26] and have confidence that the small (compared to the examined needle lengths) cut-off of the parts hanging freely in air for the low number of only 7.4% of the examined needles does not have any major impact on the reported findings.

The performed bending analysis itself was based on a semi-automatic reconstruction, which yielded inaccuracies $\Updelta e_{auto-\textit{manual}}$ even lower than intra-observer variability $\Updelta e_{vari}$. The latter was determined as 0.21 ± 0.11 mm, which was smaller than the corresponding results of 0.49 ± 0.30 mm obtained for breast brachytherapy [37]. However, it has to be noted that the latter case deals with flexible, bent catheters, and the corresponding results are thus expected to be larger. Furthermore, it has to be mentioned that the accuracy of both semi-automatic and clinical reconstruction as well as particularly that of the needle tip detection conducted in the present work is generally affected and limited by the point spread function (i.e., the resolution) of the underlying CT scans, the sampling theorem, and partial volume effects. Nevertheless, the semi-automated approach was considered to be of high quality and improved compared to previously reported needle segmentation methods utilizing MRI [38] or ultrasound [39], with median accuracies of 0.29 mm or 0.77 mm, respectively. Based on the reconstructions, we found that longer needles were (as expected) associated with larger Euclidean distances between predicted and actual tips. This has also been described in previous phantom studies [31–33] on needle deflections in prostate brachytherapy, which found deflections of up to 4.5–8 mm at maximum. In particular, depending on the needle length, we found tip deviations $\Updelta b_{\textit{trans}}$ of up to 19.9 mm for the transverse and $\Updelta b_{long}$ up to 7.8 mm for the longitudinal direction. However, there were only small differences in the calculated bending angles between the various needle lengths. Therefore, a slight trend of an increased bending for longer needles could only be assumed (e.g., considering the median bending angles provided in Table 1 of 1.7° for 100–120 mm, 1.8° for 140–160 mm, 1.9° for 160–180 mm, and 2.2° for 200–220 mm needle length) but not definitely confirmed by our data, also taking the corresponding measurement ranges of the results (Table 1) into account. Instead, the increased distances between actual and predicted needle tips for longer needles seemed to originate mainly from basic elementary geometry (intercept theorem: given a fixed angle, the distance between two lines increases as the distance from their intersection increases). In this respect, using the shortest possible needles appeared to be associated with clear benefits regarding a reduction in needle tip deviations. Furthermore, only small differences in needle bending were observed regarding the various segment zones indicated in Fig. 3b, with an increased median in the lateral zones 2 and 5. However, as the distribution of the results found for the individual segments varied also only slightly (Fig. 5c), no substantial location dependency could be defined. It has to be noted that in this respect, no valid conclusion could be drawn for segment 6, due to the small number of only eight needles implanted in this zone.

We have thus shown that the transverse needle deviations $\Updelta e_{\textit{trans}}$ occurring in the clinical workflow have a median of 1.4 mm, whereas the needle paths predicted would deviate by a median of 5.1 mm ($\Updelta b_{\textit{trans}}$) from the actual locations. Relying only on the considered external prediction approach for performing implantations could therefore result in the needles deviating even more from their intended positions than is currently the case. This is since needle bending in situ cannot be detected by considering only distal needle ends, meaning that if a needle bends, the prediction of the needle would not be accurate. To overcome this issue, external approaches such as, e.g., optical tracking would thus have to be combined with different modalities such as fluoroscopy, ultrasound [34], electromagnetic tracking [33, 40, 41], or force sensors [42] to “rectify” and correct needle courses for the transverse bending. Several studies and bending models have already shown that in this way, substantial prediction improvements regarding prostate brachytherapy phantoms are feasible [34, 35, 40, 42, 43]. A transfer to gynecologic brachytherapy should therefore be investigated. One possibility could also be to implant needles to a certain depth only, to determine and correct the bending, and to predict the tips based on this for the last few centimeters to be implanted only. This would reduce the impact of needle bending on needle predictions from outside the patient. Especially the latter workflow also provides an opportunity to ensure that the needle is implanted at the intended location relative to the HR-CTV and OARs, as relying on external predictions alone could potentially result in an unintended spatial deviation of the needle position relative to the tissue due to tissue tension or deformation during the insertion process. In this respect, it should also be mentioned that if needles bend, the same force causing the bending will also affect the tissue (even though this may be hard to detect in practice), potentially also resulting in anatomical deformations impacting the implantation procedure. However, in the present analysis it was, based on the available data, not possible to retrospectively investigate the extent of corresponding tissue deformations, so that this question remains the subject of future research. In each case, the clinical value of all these proposed methods regarding reduced effort by avoiding repetitive CT scans during interventions will have to be explored. Again, it has to be mentioned that any method predicting needle paths in situ from outside the patient body might be affected by the reported needle bending in situ to a certain extent, and we therefore think that the present work might be of value for considering implementation of external prediction approaches in general.

Our study was limited to titanium needles, which we use as default metal needles in our workflow. The effects of bending for needles made of other materials, e.g., of stainless steel or plastic, were not examined. However, for plastic needles, predicting courses in situ appears impossible, due to the associated high flexibility and non-rigidity between distal and proximal needle parts. Furthermore, our study was not only limited to our specific choice of applicator and template, but all patients studied were also implanted in our single institution by a limited number of senior physicians. Therefore, our results are specific to our current institutional situation and policies, making it impossible to determine the generality of our findings. In addition, we did not perform an assessment of the dosimetric impact of the observed needle bending. This was because brachytherapy treatment planning at our institution is performed after the implantation procedure based on the created implant. Hence, calculating a treatment plan for a simulated situation (with corrected, unbent needles) for comparison with the actual treatment plan (which was based on the bent needles) would result in different dwell position and dwell time settings of the afterloader source, making the situations hard to compare. Nevertheless, we believe that our work provides important insights into the described field of research that may help to advance the described applications in the future.

In summary, we determined the impact of needle bending on the corresponding needle paths in-situ and analyzed the needle path deviations between actually implanted and intended needle locations. We found that predicting brachytherapy needles for providing benefits regarding an improved implantation accuracy is substantially affected by transverse needle bending in situ. Future investigations should assess the clinical value, feasibility, and usability of combining prediction approaches such as optical tracking with other modalities such as fluoroscopy, if intended to be used for implantation guidance.

## Data Availability

The data that support the findings of this work are available from the corresponding author upon reasonable request.
